# Age-Related Electroencephalographic Delta and Alpha Oscillations During Sedation with Target-Controlled Propofol Infusion

**DOI:** 10.3390/jcm14093024

**Published:** 2025-04-27

**Authors:** Yeonsu Kim, Jiho Park, Woosuk Chung, Yumin Jo, Chahyun Oh, Boohwi Hong, Seongeun Kim

**Affiliations:** 1 Department of Applied Artificial Intelligence, Seoul National University of Science and Technology, Seoul 01811, Republic of Korea; yeonsu3200@gmail.com; 2 Department of Anesthesiology and Pain Medicine, Chungnam National University College of Medicine, Daejeon 35015, Republic of Korea; jihopark@cnuh.co.kr (J.P.); woosuk119@gmail.com (W.C.); lemonny87@naver.com (Y.J.); ohchahyun@gmail.com (C.O.); 3 Department of Anesthesiology and Pain Medicine, Chungnam National University Sejong Hospital, Sejong 30099, Republic of Korea; 4 Department of Anesthesiology and Pain Medicine, Chungnam National University Hospital, Daejeon 35015, Republic of Korea

**Keywords:** aging, anesthesia, EEG, spectral power, propofol, target-controlled infusion

## Abstract

**Background/Objectives**: Previous studies have reported decreases in delta and alpha power with aging under propofol anesthesia, often confounded by reduced target concentrations in older patients. This study aimed to investigate electroencephalography (EEG) dynamics under propofol sedation using target-controlled infusion (TCI) while maintaining consistent effect-site concentrations across age groups. **Methods**: We conducted a comparative observational study of 44 patients scheduled for orthopedic upper extremity surgery under regional anesthesia. Patients were categorized into the younger (20–39 years, *n* = 23) and older (50–69 years, *n* = 21) age groups. EEG data were recorded from four frontal electrodes, with a specific focus on delta and alpha frequency bands, while the effect-site concentration of propofol was maintained at 3.0 μg/mL using TCI. **Results**: TCI-adjusted propofol administration with the same target concentration results in different total drug delivery between the two age groups, according to age-related pharmacokinetic differences. The younger age group exhibited higher delta power, indicating an age-associated decline. Alpha power remained stable across age groups despite the differences in drug delivery, while older patients demonstrated decreased frontal alpha synchronization, highlighting age-related changes in brain connectivity. **Conclusions**: This study demonstrates that delta power decreases with age, even under standardized propofol concentration, while alpha power remains consistent, suggesting its possibility as an indicator of sedation depth. In contrast, the variations in delta power and alpha connectivity in different age groups suggest the need for age-specific anesthesia dosing to enhance safety and efficacy. Therefore, these findings contribute to a better understanding of age-related neurophysiological responses to anesthesia.

## 1. Introduction

Electroencephalography (EEG) is a valuable biomarker for reflecting the brain’s complex neurophysiological dynamics, making it indispensable in understanding the effects of anesthesia [1,2,3]. Processed EEG metrics, such as the bispectral index (BIS) and patient state index (PSI), have contributed to advancements in anesthesia management; however, their reliability remains under active investigation [4,5,6]. These advancements have led not only to reduced anesthetic consumption but also to a decreased risk of intraoperative awareness and postoperative cognitive dysfunction [7].

GABAergic anesthetics, including propofol, are strongly associated with anesthesia-induced unconsciousness through the modulation of delta (0.5–4 Hz) and alpha (8–13 Hz) oscillations [8,9]. The emergence and distribution of these oscillations are known to change with age [10,11,12,13,14]. During the first year of life, delta power increases as the brain develops, accompanied by the anteriorization of alpha oscillations, which become more coherent [10,11]. In contrast, aging is typically associated with declines in both delta and alpha power [12,13,14]. However, in clinical practice, anesthetic dosages are often substantially reduced for older patients to account for age-related changes in drug metabolism and sensitivity [13,14]. These adjustments may confound the relationship between age and EEG patterns. Although previous studies have suggested that reductions in EEG power with age may be attributed to neurophysiological aging, many of these studies lacked standardized dosing protocols that properly accounted for age-related variability. In particular, anesthetic dosages varied considerably among elderly patients, making direct comparisons challenging [15,16,17]. Due to a lack of standardization, the observed changes in EEG dynamics may reflect variations in anesthetic dosing rather than intrinsic neurophysiological aging.

Target-controlled infusion (TCI) systems can address these challenges by offering personalized anesthesia management. TCI with the Schnider model automatically adjusts anesthetic delivery based on individual characteristics such as age, weight, height, and sex [18,19]. By maintaining consistent effect-site concentrations tailored to the individual characteristics of each patient, TCI can mitigate dosage-related variability, allowing for a more precise evaluation of the relationship between age and EEG dynamics.

This study aims to address these limitations by investigating EEG dynamics in two age groups (younger: 20–39 years; older: 50–69 years) under standardized propofol sedation. Using TCI with the Schnider model to maintain a constant effect-site concentration of 3.0 μg/mL for both groups, we analyzed delta and alpha frequency bands to assess age-related differences. To minimize confounding from surgical pain artifacts, all patients underwent regional anesthesia via brachial plexus block (BPB). By controlling anesthetic delivery to achieve the same effect-site concentration, this study seeks to provide clearer insights into the neurophysiological effects of aging on brain activity during anesthesia.

## 2. Materials and Methods

### 2.1. Ethics Approval

This is a sub-analysis of a prospective study on EEG collection under sedation. The primary study was approved by the Institutional Review Board of the Chungnam National University Hospital, Korea (approval number: CNUH 2020-10-071; date: 2 December 2020) and was preregistered on the Korean clinical trial registry (KCT0005848). All patients received detailed information about the study and signed written informed consent forms. The study was conducted in accordance with the Declaration of Helsinki.

### 2.2. Patient Population

**Figure 1:**
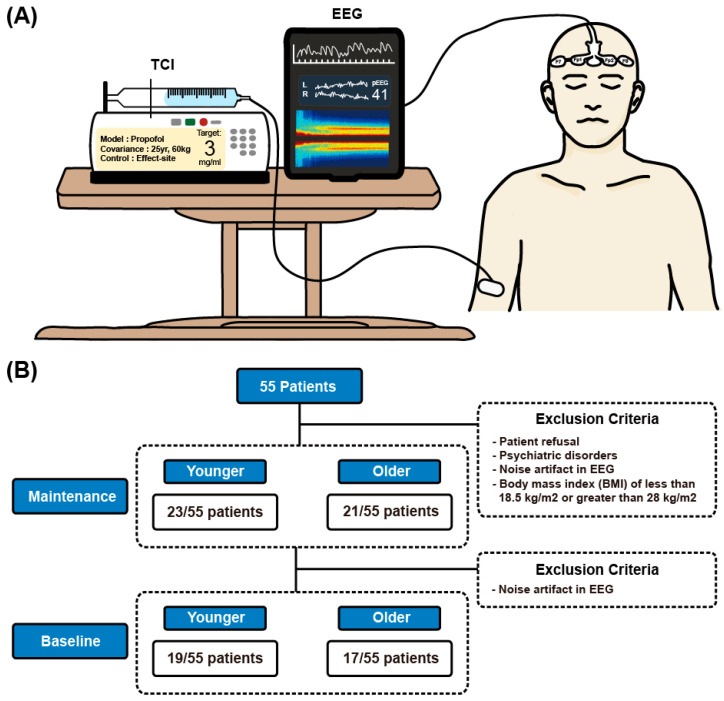
**Study design for electroencephalographic (EEG) analysis during propofol sedation.** (**A**) Diagram of the data collection process. EEG analysis was performed in patients who received propofol sedation with an effect-site target of 3.0 μg/mL using target-controlled infusion (TCI). EEG recordings were taken from channels Fp1, Fp2, F7, and F8. (**B**) EEG data from 55 patients were analyzed. Clean EEG epochs were visually extracted from 23 younger and 21 older adult patients during sedation and from 19 younger and 17 older adult patients during the baseline phase. pEEG, processed electroencephalography.

Participants included adult patients scheduled for orthopedic upper extremity surgery with BPB at the Chungnam National University Hospital. The inclusion criterion was adults aged 20–69 years who required surgery between March 2021 and March 2022. For comparative analysis, participants were categorized into the younger (20–39 years) and older (50–69 years) age groups. By selecting participants aged 20–39 and 50–69, we aimed to contrast younger and older adults while minimizing overlap in age-related physiological changes. Including individuals aged 40–49 could have introduced intermediate variables that might confound the clear comparison between distinctly younger and older groups. Initially, 55 patients were recruited for the study. Exclusion criteria were patient refusal, uncontrolled diabetes mellitus and hypertension, significant cardiopulmonary disease, psychiatric disorders, stroke or cerebrovascular disease, renal dysfunction, neuronal disease, and body mass index (BMI) of <18.5 or >28 kg/m^2^. Ultimately, 44 patients were included in the final analysis, with 23 and 21 in the younger and older age groups, respectively (Figure 1B). All patients were clinically stable on the day of the study.

### 2.3. Anesthetic Management

All brachial plexus blocks (BPBs) were performed under ultrasound guidance using a 1:1 mixture of 0.75% ropivacaine and 1% lidocaine without any adjuvants. Intraoperative monitoring included non-invasive intermittent blood pressure measurement, a three-lead electrocardiogram, and pulse oximetry. SedLine^®^ four-channel EEG sensors (Masimo, Irvine, CA, USA) were attached to the foreheads of all patients for continuous EEG monitoring. After confirming the establishment of surgical anesthesia, sedation was initiated using propofol administered by TCI based on the Schnider model [18]. Propofol was delivered through the Module DPS TCI system (Fresenius Kabi, Bad Homburg, Germany), which was connected to a peripheral intravenous line with a 15 cm extension. Oxygen was supplied at 5 L/min through a mask to ensure adequate oxygenation. The primary effect-site concentration of propofol was set at 3.0 μg/mL based on its established efficacy in achieving adequate sedation and patient comfort during surgical procedures while minimizing the risk of apnea. This concentration provides a sufficient depth of sedation to ensure complete patient immobility and comfort while maintaining spontaneous respiration. All patients maintained spontaneous breathing throughout the procedure, and no episodes of respiratory depression requiring intervention were observed in either age group. Figure 1A illustrates the experimental procedure.

### 2.4. Data Acquisition

We analyzed EEG data recorded from frontal channels (Fp1, Fp2, F7, and F8) using the SedLine^®^ four-channel EEG sensors. The display settings on the SedLine^®^ devices were uniformly standardized at 30 mm/s and 25 µV/mm for paper speed and amplitude, respectively, with a sampling rate of 178 Hz to ensure consistency in data visualization and interpretation. All intraoperative EEG and propofol infusion data were obtained from the prospective registry of intraoperative clinical information for surgical patients at Chungnam National University Hospital, using a free data collection program, Vital Recorder (version 1.8, accessed at https://vitaldb.net, Seoul, Republic of Korea) [20]. EEG recordings were subsequently converted to European Data Format (EDF) files from vital recorder files. Baseline EEG data were recorded in the waiting room 10 min before entering the operating room, using a setup similar to that of the intraoperative EEG. Each patient was instructed to lie still on a bed with eyes closed, and EEG data were continuously recorded for 5 min without any movement to ensure a stable and clear baseline recording. Finally, the baseline EEG data were exported as EDF files from the Masimo SedLine monitors for further analysis.

### 2.5. EEG Analysis

#### 2.5.1. Data Preprocessing

Preprocessing of EEG data was conducted using MATLAB (MathWorks, Natick, MA, USA). Continuous EEG recordings were started upon entry to the operating room, and EEG data from the onset of anesthesia were extracted for analysis. For analysis, EEG segments were selected at 10, 20, and 30 min after the initiation of anesthesia. A 3-min epoch of artifact-free EEG data was identified and extracted at each time point. Baseline EEG data were recorded 10 min prior to entering the operating room, with a 100 s segment of artifact-free data selected for analysis. Visual inspection was employed to ensure that all selected segments were artifact-free, guaranteeing reliable and comparable preoperative and intraoperative assessments.

#### 2.5.2. Spectral Analysis

Spectral analysis was performed using the multitaper method, implemented with the Chronux toolbox. Multitaper parameters included a 4 s window length (T = 4 s) with no overlap, a time–bandwidth product of 3 (TW = 3), and the employment of five tapers (K = 5), yielding a spectral resolution of 1.5 Hz. For each group, group-averaged spectrograms and spectra were produced by calculating the median of the data across the study population. The interquartile range (IQR) was plotted as shaded areas within the group median spectra, providing a visual representation of data distribution. To map the spatial distribution of frequency band power across the frontal scalp, median power spectra were calculated for each 3 min epoch using electrodes Fp1, Fp2, F7, and F8. Subsequently, these power spectra were averaged for the delta (0.5–4 Hz) and alpha (8–13 Hz) frequency bands. This visualization was generated using the topoplot function in the FieldTrip 2019 software package [21]. All spectral analyses were conducted with custom MATLAB codes, facilitating precise control over data processing and analysis procedures.

#### 2.5.3. Coherence Analysis

We used coherence analysis to quantify the synchrony between two EEG signals within specific frequency bands. This analysis estimated coherence and coherograms between bipolar frontal left (F7–Fp1) and right (F8–Fp2) electrode pairs using the multitaper method in the Chronux toolbox in MATLAB 2023b [22]. Coherence analysis followed the same multitaper parameters as spectral analysis (see Spectral Analysis subsection). Group-averaged coherence values were derived by calculating the median across all patients within each age cohort. This analysis was performed separately for the delta (0.5–4 Hz) and alpha (8–13 Hz) frequency bands to clarify specific spectral characteristics.

#### 2.5.4. Functional Connectivity Analysis

We extended our analysis to include the weighted Phase Lag Index (wPLI), a sophisticated measure of phase synchronization between EEG signals across different scalp regions, to improve our understanding of the neurophysiological effects of propofol sedation [23]. The wPLI is designed to overcome the limitations of volume conduction by focusing on the asymmetry of phase differences, thereby providing a more accurate representation of true neuronal connectivity. The detailed formulation is described in Appendix A.2. This formulation emphasizes phase leads and lags (positive and negative phase differences) while discounting phase differences around zero, thereby highlighting genuine connectivity over artifactual coherences. Additionally, this wPLI analysis allowed us to better understand the effects of propofol on brain network interactions and the differences in connectivity patterns between age groups.

### 2.6. Statistical Analysis

We implemented statistical techniques described in the literature using custom MATLAB codes (MathWorks, Natick, MA, USA) to enable statistical inference and group-level analysis [24]. The Shapiro–Wilk test was employed to assess the normality of the distribution of the variables. Normally and non-normally distributed variables were expressed as mean ± standard deviation and median and IQR, respectively. To evaluate normally distributed variables, an independent Student’s *t*-test was used to compare the means between groups, while the Wilcoxon rank-sum test was applied for non-normally distributed variables. Categorical variables were expressed as counts and percentages and were analyzed using Fisher’s exact test.

In the quantitative analysis of EEG data, we evaluated statistically significant spectral differences between the two groups for each channel. Frequency band power values between groups were compared employing the nonparametric Wilcoxon rank-sum test, and *p*-values were subsequently calculated. To mitigate the risk of type I error due to multiple comparisons, *p*-values were adjusted using the Benjamini–Hochberg procedure to control for false discovery rate (FDR) [25]. This FDR correction was applied across the range of *p*-values obtained from analyzing the four-channel EEG data. Between-group differences were considered statistically significant when the FDR-adjusted *p*-values were <0.001. The rigorous application of these statistical methods ensured the robustness and reliability of the study findings.

For a detailed examination of age-related differences in EEG power at specific time points, we conducted time-specific mixed-effects analyses [26]. For each time point (10, 20, and 30 min), we fitted a linear mixed-effects model with the group as a fixed effect and the subject as a random effect. The model was specified as follows:

Power~group + (1|subject)

where Power represents either alpha or delta power, the group is the categorical variable indicating age group (younger vs. older), and (1|subject) accounts for the repeated measures within subjects. This analysis allowed us to evaluate the group differences at each specific time point while accounting for individual variability. For each frequency band (alpha and delta), we analyzed the impact of age group at each individual time point (10, 20, and 30 min). The statistical significance was assessed using F-tests, with *p* < 0.05 considered statistically significant. All analyses were performed using MATLAB R2021b (MathWorks, Natick, MA, USA) with the Statistics and Machine Learning Toolbox.

## 3. Results

We analyzed multichannel frontal EEG data from patients in the younger (*n* = 23, median age: 27 years) and older (*n* = 21, median age: 60 years) age groups who underwent elective upper extremity surgery under regional anesthesia and propofol sedation. Figure 1B shows the specifics of participant selection. Table 1 summarizes the clinical characteristics of the eligible patients. No significant differences were found between the younger and older groups except for height. Over 87% of the participants in each group were classified as having American Society of Anesthesiologists (ASA) class II physical status. Throughout the 30 min interval, patients in the younger age group received significantly higher doses of propofol than those in the older age group, as controlled via TCI with the Schnider model, to achieve the same effect-site concentration of 3.0 μg/mL.

### 3.1. Study Design and TCI Management

**Figure 2:**
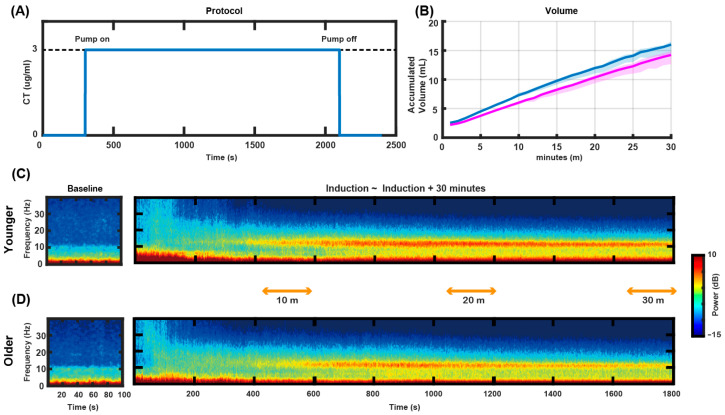
**Propofol administration and its effects on electroencephalographic (EEG) spectral power dynamics.** (**A**) Illustration of the target-controlled infusion (TCI) protocol specifying concentration at the target site (CT) of 3.0 μg/mL. (**B**) Graphical representation of accumulated propofol doses in the younger (blue) and older (pink) age groups over 30 min. (**C**,**D**) EEG power spectrogram within the 0–40 Hz frequency range for patients in the younger and older age groups, showing dynamic changes at baseline and 10, 20, and 30 min intervals after propofol infusion.

**Figure 3:**
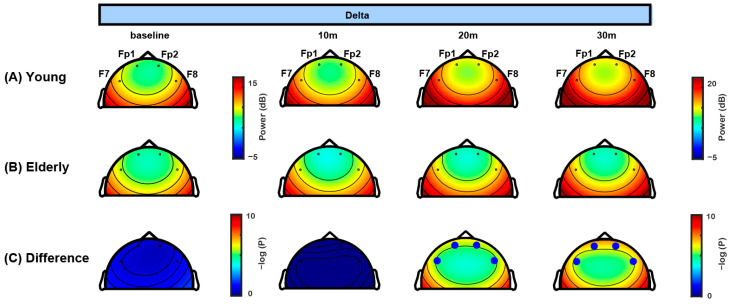
**Topographic variations in delta band power during sedation.** (**A**) Topographic maps of delta power for the younger age group at baseline and 10, 20, and 30 min intervals after initiating propofol infusion, indicating a progressive increase in delta power, particularly at later time points. (**B**) Topographic maps of delta power for the older age group at identical time points, demonstrating consistent delta power. (**C**) Statistical comparison of delta power between the two age groups at each interval, using negative log-transformed *p*-values (−log(*p*)) to denote statistical significance. Large blue markers indicate statistically significant differences (*p* < 0.001). A marker has been added to the color bar to indicate the threshold for statistical significance. Significant age-related differences in delta power between age groups become evident at 20 and 30 min intervals, reaching a threshold of *p* < 0.001.

**Figure 4:**
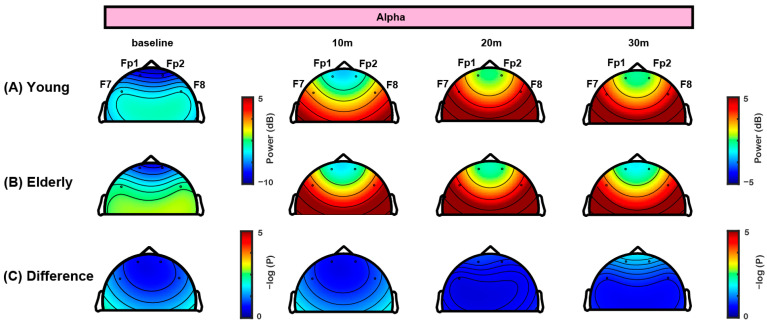
**Topographic variations in alpha band power during sedation.** (**A**) Topographic maps of alpha power for the younger age group at baseline and 10, 20, and 30 min intervals after initiating propofol infusion, indicating a progressive increase in alpha power, particularly at later intervals. (**B**) Topographic maps of alpha power for the older age group at identical time points, showing a similar distribution of alpha power compared to that of the younger age group. (**C**) Statistical comparison of alpha power between the two age groups at each interval, using negative log-transformed *p*-values (−log(*p*)) to denote statistical significance. The analysis reveals no significant differences in alpha power between age groups at 20 and 30 min intervals, demonstrating uniformity in alpha power between different age groups during anesthesia.

In this study, propofol TCI was set with an effect-site concentration of 3.0 μg/mL for all patients, as illustrated in Figure 2A, contrasting with previous studies that adjusted TCI concentrations 20–50% lower for elderly patients compared to the younger group [15,16,17]. All patients were managed under a uniform anesthesia protocol, ensuring standardized administration via TCI with the Schnider model. Figure 2B presents the doses of propofol administered for the two age groups, showing that patients in the older age group received a lower total dose of propofol to maintain the target concentration than those in the younger age group. The difference in accumulated infusion volume between the groups became more pronounced with increasing duration of anesthesia. Baseline EEG data revealed activated delta waves and minor alpha activity in both age groups (left panel in Figure 2C,D). Statistical analysis indicates that while the alpha power did not significantly differ between the two groups, the delta power significantly differed (Figure 3 and Figure 4). Further detailed analysis follows.

### 3.2. Frontal Topographical Spectral Power Analysis

The frontal topographical spectral power of the delta and alpha frequency bands was analyzed at baseline and 10, 20, and 30 min intervals (Figure 3 and Figure 4). Figure 3 presents the progression of the delta spectral power over time. In the younger age group (Figure 3A), the delta power significantly increased as anesthesia progressed, particularly at the 20 and 30 min intervals. In contrast, the older age group exhibited consistent delta power at all time points (Figure 3B). A statistical comparative analysis between the two groups revealed substantial disparities, with significant differences (*p* < 0.001) observed at the 20 and 30 min intervals in all four channels (Figure 3C), which was also confirmed by mixed-effects analysis (Table A1). These results indicate a pronounced age-related variation in delta spectral power, with the younger age group showing a marked increase in delta power compared to the older age group. Figure 4 illustrates the frontal topographical spectral power of the alpha frequency band. Both the younger and older age groups showed strong alpha spectral power. Alpha power gradually increased over time in the younger age group (Figure 4A) but remained relatively stable at all time points in the older age group (Figure 4B). Despite this increase in the younger age group, a comparative analysis did not show significant differences in alpha spectral power between the two groups (Figure 4C). Mixed-effects analysis supported these findings, showing no consistent group differences in alpha power across time points (Table A1).

### 3.3. Frontal Connectivity Analysis

**Figure 5:**
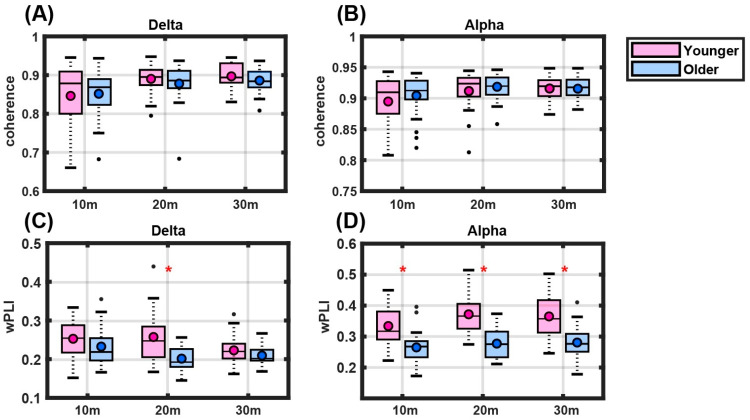
**Frontal connectivity during sedation.** (**A**) Box plots of delta band coherence at intervals of 10, 20, and 30 min after initiating propofol infusion, revealing no significant differences between the younger and older age groups. (**B**) Box plots of alpha band coherence at identical intervals, indicating no significant age-related differences in the coherence analysis. (**C**) Box plots of delta band weighted phase lag index (wPLI) at 10, 20, and 30 min after initiating propofol infusion, revealing a significant difference at the 20 min mark, with the older age group showing altered connectivity. (**D**) Box plots of the alpha band wPLI, demonstrating a consistent and significant decrease in connectivity for the older age group at all time points assessed. Statistical significance is marked with a red asterisk at *p* < 0.001. The dots within the box plots represent individual subject data points, with outliers indicated as separate markers.

We examined the coherence within the frontal regions in different age groups during sedation by computing the median coherence using bipolar electrode configurations F7–Fp1 and F8–Fp2 (Figure 5A,B). Regarding the analysis of specific frequency bands, the coherence levels of delta (0.5–4 Hz) and alpha (8–13 Hz) were evaluated at 10, 20, and 30 min intervals after the initiation of propofol administration. The median coherence levels for these intervals were compared between the younger and older age groups, and the results are presented in box plots for the delta (Figure 5A) and alpha (Figure 5B) frequency bands. Our results revealed no significant differences between the two groups in the delta or alpha frequency bands at any of the time points analyzed. Notably, coherence values for the delta and alpha oscillations were mainly >0.8. There is a high similarity between channels, which may be attributed to volume conduction. Therefore, we performed an additional analysis using the wPLI to minimize the impact of volume conduction.

The analysis of the wPLI within the delta and alpha frequency bands indicated age-related differences in frontal region connectivity during sedation. Specifically, a significant difference in connectivity was observed in the delta frequency band only at the 20 min interval after the induction of anesthesia (*p* < 0.001), with the older age group showing altered synchronization compared to the younger age group (Figure 5C). However, no consistency was found across time points. In the alpha frequency band, the older age group exhibited a significantly lower wPLI at all measured time points (10, 20, and 30 min) (Figure 5D). This consistent reduction in connectivity suggests a decrease in alpha-band synchronization in the frontal regions in the older age group throughout the assessment period. The significant findings (*p* < 0.001) highlight the impact of aging on neural synchronization. While the coherence levels in the delta and alpha frequency bands did not significantly differ between the age groups, the wPLI analysis demonstrated age-related connectivity differences. Here, connectivity refers to the overall network structure, while synchrony indicates the temporal coordination of neural oscillations.

## 4. Discussion

Our study investigated the age-related effects of propofol-induced sedation on EEG dynamics using a TCI Schnider protocol, focusing on the delta and alpha frequency bands. Our key findings include the following when maintaining a fixed target concentration of 3.0 μg/mL across age groups using TCI’s automatic dose adjustment: (1) Alpha band power was comparable between younger and older patients. (2) Delta band power showed significant age-related reductions in the older group, reflecting its sensitivity to aging. (3) Alpha band connectivity, assessed via wPLI, declined significantly in older patients, suggesting reduced frontal coherence with age.

### 4.1. Frontal Alpha Band Power and Age

Previous studies typically reduce target concentrations by approximately 20–50% in elderly patients compared to younger adults [15,16,17]. However, our study maintained a consistent target concentration of 3.0 μg/mL across all age groups using TCI’s automated pharmacokinetic adjustments. Despite receiving a lower total dose of propofol, the older group exhibited alpha power levels comparable to those of the younger group. This suggests that earlier observations of reduced alpha power with age may result more from the routine practice of lowering target concentrations in elderly patients than from aging itself [15,16]. Our controlled TCI protocol also ensured that older patients received significantly less propofol than younger individuals at the same target concentration (Figure 2B), as infused by the Schnider model, which accounts for age-related reductions in metabolic rate and increased brain sensitivity to propofol [17].

To further validate these findings for the elderly group (aged > 60 years), we conducted supplementary analyses comparing young adults (20–39 years) with elderly patients aged 60–69 years (*n* = 12). Although the smaller sample size in this subgroup reduced the statistical power, the results presented in Table A2 confirmed no significant differences in alpha band power between the two groups, as determined by mixed-effects analysis. This finding strengthens our conclusion that TCI’s automatic dose adjustment effectively maintains consistent alpha oscillations for a narrower elderly age range. In contrast, delta power differences remained significant in this supplementary analysis (*p* < 0.001), consistent with our primary findings, indicating that delta power may be more sensitive to age-related changes.

### 4.2. Thalamocortical Circuit and Frontal Alpha Connectivity

Our findings indicate that propofol anesthesia typically increases alpha power and coherence in the frontal region [2,3,8,27]. Mathematical modeling of physiological EEG dynamics suggests that interactions between the thalamus and frontal cortex generate oscillatory activities in the alpha band, leading to high frontal coherence [28,29]. Alpha coherence during anesthesia decreases with aging since older patients cannot maintain stable thalamocortical synchronization [12].

Our study showed that the alpha band power remained comparable between younger and older age groups when propofol was maintained at a constant effect-site concentration using TCI with the Schnider model. However, the older age groups exhibited lower frontal connectivity during propofol-induced sedation than the younger age group. Frontal alpha coherence is closely linked to the thalamus, which typically begins to atrophy around the ages of 50 and 60 years [30,31]. Unlike the cerebral cortex, the thalamus exhibits limited plasticity and compensatory ability after damage, potentially impairing its role as a central hub for integrating sensory and motor signals [32]. This reduced capacity may explain why early stages of thalamic atrophy do not necessarily manifest as significant changes in alpha band power. However, wPLI network analysis, which assesses functional connectivity through phase synchronization, detected subtle disruptions in the alpha band, potentially reflecting the weakening thalamocortical feedback between the thalamus and cortex in older patients. These findings underscore the importance of connectivity analysis in identifying age-related brain changes.

Our results align with those of several previous studies indicating that healthy aging does not significantly affect alpha power between age groups, although functional connectivity tends to decrease in older adults [33,34,35]. Decreased alpha power in EEG has been linked to neurological conditions, such as Alzheimer’s disease, delirium, and other cognitive impairments [36,37], suggesting that alpha power changes in older individuals under anesthesia may reflect underlying cognitive conditions rather than normal aging. Our findings indicate that anesthetic dosing plays a crucial role in alpha power modulation. While lower doses might contribute to reduced alpha power, excessive anesthetic levels are also associated with suppressed alpha oscillations. This suggests a complex dose-dependent relationship requiring further study. Therefore, cognitive function and anesthetic dosage should be considered when assessing EEG patterns in older patients during anesthesia.

Our findings indicate that impaired alpha connectivity, reflecting weakened thalamocortical synchronization, may serve as a biomarker for increased susceptibility to postoperative delirium (POD). Integrating alpha connectivity metrics into sedation monitoring devices, such as the SedLine^®^ system, could enable clinicians to identify patients at elevated risk for POD. This real-time monitoring would allow for the implementation of preventive strategies, such as adjusting anesthetic depth to avoid burst suppression patterns, thereby enhancing patient safety and outcomes.

### 4.3. Frontal Delta Band Power and Age

Compared to the alpha band power, the delta band power exhibited significant age-related variations, highlighting the importance of considering age for accurate EEG evaluations. The delta band power, which reflects neural activity primarily in the cerebral cortex [38,39], decreases as cortical gray matter declines, which begins in the mid-20s and accelerates with age [40,41,42]. This reduction in the delta band power becomes more pronounced with aging, despite using TCI’s automatic dose adjustment to maintain consistent effect-site concentrations.

Our findings indicate that the observed reduction in delta power among older patients during propofol sedation may serve as a biomarker for increased cortical sensitivity to the anesthetic. Integrating real-time delta power monitoring into sedation protocols could enable anesthesiologists to tailor propofol dosing more precisely for elderly patients, minimizing the risk of over-sedation and associated complications. For instance, TCI systems could incorporate delta power metrics to adjust infusion rates dynamically, ensuring optimal sedation depth while accounting for age-related neurophysiological changes. Such an approach could enhance patient safety and efficacy of sedation by providing a more individualized anesthetic management strategy.

However, our study found that frontal wPLI connectivity in the delta oscillations remained unchanged. The widely recognized absence of delta coherence during propofol-induced unconsciousness is attributed to the disruption of thalamocortical and corticocortical interactions, along with the rapid fragmentation of neuronal networks [2,9]. These mechanisms likely explain the reduced synchrony of delta oscillations and the lack of significant differences between age groups observed in our study. This finding suggests that frontal delta connectivity may not be closely associated with age during anesthesia. Developing age-specific anesthesia protocols that reflect these neural dynamics could improve EEG monitoring accuracy and enhance patient outcomes.

### 4.4. Study Limitations

Our study has several limitations. First, although we observed significant differences in the delta band power but not in the alpha band power between these two groups, the relatively small sample size of 44 patients restricts the generalizability of our findings. Furthermore, the participant age range was restricted to individuals aged 20–39 and 50–69 years. Future research should increase the sample size and include patients over 70 years, a demographic more likely to have mild cognitive impairment and potentially distinct EEG responses to propofol, which would provide a more comprehensive understanding of propofol’s effects on EEG patterns. Second, our study focused on propofol with a fixed sedation level. Investigating the effects of other anesthetic agents or varying sedation depths could help validate and expand our findings, improving the applicability of our results to a wider range of clinical scenarios. Third, our analysis was primarily based on frontal EEG recordings, providing a limited view of the brain’s overall neuronal activity. Future studies using multichannel EEG electrodes could provide a more comprehensive perspective on neural dynamics, enriching our understanding of how different brain regions respond to anesthesia.

## 5. Conclusions

Our findings suggest that age-related TCI-adjusted dosing may be important for accurately assessing EEG changes associated with aging. In contrast to previous studies, we observed no significant differences in alpha power between young and elderly groups when propofol dosing was carefully controlled. Additionally, we noted a potential decrease in delta power and reduced alpha connectivity in the elderly. These observations indicate that appropriate dosage adjustment could be a key factor in elucidating age-related neurophysiological changes and may contribute to more personalized and effective strategies in clinical anesthesia practice.

## Figures and Tables

**Table 1:** Demographic and clinical characteristics of patients.

	Younger Age Group (*n* = 23)	Older Age Group (*n* = 21)	*p*-Value
Age (years), median (IQR)	27 (22–34)	60 (58–65)	-
Height (cm), mean (SD)	170.9 (7.5)	161.2 (8.9)	<0.001
Weight (kg), mean (SD)	68.7 (11.6)	64.7 (12.2)	0.279
BMI (kg/m^2^), mean (SD)	23.5 (3.3)	24.7 (2.9)	0.205
Sex (male), N (%)	17 (73.9)	10 (47.6)	0.121
ASA I, N (%)	0 (0)	2 (0.09)	1.0
ASA II, N (%)	20 (0.87)	19 (0.91)	
ASA III, N (%)	3 (0.13)	0 (0)	
PSI, median (IQR)	35 (31–49)	36 (30–48)	0.547
Dose of propofol at 10 min (mL), median (IQR)	7.3 (6.9–7.6)	6.1 (5.8–6.5)	<0.001
Dose of propofol at 20 min (mL), median (IQR)	12.1 (11.7–12.9)	10.4 (9.9–11.6)	<0.001
Dose of propofol at 30 min (mL), median (IQR)	16.0 (15.5–17.6)	14.3 (13.6–16.3)	<0.001

Values are expressed as median (IQR), mean (SD), or number (percentage). BMI, body mass index; IQR, interquartile range; SD, standard deviation; PSI, Patient State Index; ASA, American Society of Anesthesiologists.

## Data Availability

The data used and analyzed during the current study are not publicly available due to concerns about human subjects.

## Abbreviations

The following abbreviations are used in this manuscript:

BIS	Bispectral index
PSI	Patient state index
TCI	Target-controlled infusion
wPLI	Weighted phase lag index
BPB	Brachial plexus block

## Appendix A

### Appendix A.1

**Table A1:** Results of mixed-effects analysis for alpha and delta power across time points between younger (20–39 years) and older (50–69 years) groups.

**Alpha Power**		
**Time Point**	**F Value**	***p* Value**
10 min	0.001	0.971
20 min	0.820	0.367
30 min	4.451	0.037
**Delta Power**		
**Time Point**	**F Value**	***p* Value**
10 min	4.086	0.126
20 min	14.544	0.002
30 min	18.158	<0.001

Analysis based on linear mixed effects models comparing younger (20–39 years, *n* = 23) and older (50–69 years, *n* = 21) groups.

**Table A2:** Results of mixed-effects analysis for alpha and delta power across time points younger (20–39 years) and older (60–69 years) groups.

**Alpha Power**		
**Time Point**	**F Value**	***p* Value**
10 min	0.001	0.907
20 min	1.837	0.177
30 min	3.842	0.052
**Delta Power**		
**Time Point**	**F Value**	***p* Value**
10 min	4.534	0.035
20 min	15.42	<0.001
30 min	18.622	<0.001

Analysis based on linear mixed effects models comparing younger (20–39 years, *n* = 21) and older (60–69 years, *n* = 12) groups.

### Appendix A.2

The phase information of EEG signals used to compute wPLI was extracted using the Hilbert transform. For two signals, *X* and *Y*, the phase of each signal at every time point *t* is represented as *ϕ_X_*(*t*) and *ϕ_Y_*(*t*), respectively. The phase difference Δ*ϕ*(*t*) between these signals is calculated as follows:

Δ*ϕ*(*t*) = *ϕ_X_*(*t*) − *ϕ_Y_*(*t*)

Furthermore, wPLI quantifies the asymmetry of this phase difference distribution to minimize the impact of zero-lag interactions likely caused by volume conduction. The formula for wPLI is presented as follows:

$$wPLI=\frac{|\sum_{t=1}^{T}sign(\Delta \phi (t))\cdot |\Delta \phi (t)||}{\sum_{t=1}^{T}\left|\Delta \phi (t)\right|}$$

where *T* represents the total number of time points in the analyzed epoch, and sign(Δ*ϕ*(*t*)) extracts the sign of the phase difference at each time point.
